# Selenium Supplementation for Autoimmune Thyroiditis: A Systematic Review and Meta-Analysis

**DOI:** 10.1155/2014/904573

**Published:** 2014-12-11

**Authors:** Yaofu Fan, Shuhang Xu, Huifeng Zhang, Wen Cao, Kun Wang, Guofang Chen, Hongjie Di, Meng Cao, Chao Liu

**Affiliations:** Endocrine and Diabetes Center, Jiangsu Province Hospital on Integration of Chinese and Western Medicine, Nanjing University of Chinese Medicine, Nanjing 210000, China

## Abstract

Many studies have reported that selenium (Se) has a close relationship with autoimmune thyroiditis (AIT). The therapeutic effect of Se supplementation in AIT treatment remains unclear. The objective of the present study was to determine the efficacy of Se supplementation for the treatment of AIT. A structured literature search was undertaken to identify all randomized controlled trials conducted in patients with AIT receiving Se supplementation or placebo. Nine studies enrolling a total of 787 patients were included. The results showed that Se supplementation with duration 6 months significantly dropped the TPOAb titers but did not decrease the TgAb titers. Patients assigned to Se supplementation for 12-month duration showed significantly lower TPOAb titers and TgAb titers. Patients after Se supplementation had a higher chance to improve the mood or well-being compared with controls. Se supplementation is associated with a significant decrease in TPOAb titers at 6 and 12 months; meanwhile, the TgAb titers can be dropped at 12 months. After Se supplementation treatment, patients had a higher chance to improve the mood without significant adverse events.

## 1. Introduction

The thyroid is the organ with the highest selenium (Se) concentrations per gram among all tissue [1, 2]. Se is the only trace element to be specified in the genetic code and the main structure of it is selenoproteins, including glutathione peroxidase (GPXs), thioredoxin reductases (TRs), and iodothyronine deiodinases (DIO) [3, 4], which have been functionally characterized as having reduction of DNA damage, antioxidant processes, and hormone metabolism [5]. Se deficiency has been associated with many conditions, such as increased thyrocytes damage, infections, and the incidence of cancer [6]. Some studies have reported that Se deficiency causes a decline in GPXs and DIO enzymes activity and the concentrations of hydrogen peroxide (H_2_O_2_), which will eternally result in impairing the synthesis of thyroid hormones [7].

Autoimmune thyroiditis (AIT) is the most common human organ-specific autoimmune disease. Hashimoto's thyroiditis (HT) accounts for more than 90% of all patients. The incidence of this disease is approximately 1% in the general population, and women are ten times more often affected than men. The tendency is even more obvious at the postmenopausal period [8, 9]. Thyroglobulin antibody (TgAb) and thyroid peroxidase autoantibody (TPOAb) are the main antibodies detected in AIT. TgAb is present in high titers in sera of patients with AIT (40%–70%) [10], and TPOAb is present in the majority of AIT (>80%) [11]. Currently, several factors were reported to be associated with AIT including gene, environment, diet, and diseases. Se deficiency could induce the damage of thyroid cell and the tissue. However, it is still unknown whether Se deficiency was an important condition for AIT or marker for increased AIT incidence.

Se supplementation to improve autoimmune process has been explored. There are some inconsistent conclusions on effects of Se supplementation for treating AIT. Several papers have maintained that it had no evidences on the effects of Se supplementation, while the others suggested that there was beneficial evidence for Se supplementation, including the decrease of TPOAb and TgAb titers [12–14].

Therefore, we performed this systematic review and meta-analysis of all currently available randomized controlled trials (RCTs) to determine whether Se supplementation is an effective treatment for AIT.

## 2. Materials and Methods

### 2.1. Search Strategy

Medline, Embase, the Cochrane Central Register of Controlled Trial, Chinese Biomedical Literature Database, National Knowledge Infrastructure, WANFANG, and VIP Database were searched until 31 March 2014. The following search terms were used individually or combined: “selenium,” “sodium selenite,” “thyroid,” “thyroiditis,” “AITD,” “autoimmune thyroiditis,” and “Hashimoto thyroiditis,” with no language restriction. Two investigators (Yaofu Fan and Huifeng Zhang) independently screened all titles and abstracts to identify articles for full review. Any discrepancy was solved by discussion and consensus reached through a third author (Shuhang Xu). Only published studies with full-text articles were included in our meta-analysis.

### 2.2. Inclusion Criteria

Only the studies that met the following criteria were included: (1) RCT study sign; (2) all participants were AIT patients; (3) one group was treated with Se supplementation compared with the other groups receiving only placebo or no treatment; (4) the main outcome measures were TPOAb titers and TgAb titers.

### 2.3. Quality Assessment of Primary Studies

Two authors (Yaofu Fan and Huifeng Zhang) independently evaluated the quality of all included RCTs by Jadad scale in the following domains: randomization, blinding, and description of withdrawals and dropouts [22]. A cut score of 3 was used to indicate high quality studies as it has been reported to be sufficient to determine high quality versus low quality in previous studies.

### 2.4. Data Extraction

Two authors (Yaofu Fan and Huifeng Zhang) independently extracted data based on a predesigned data extraction form. Information was extracted on baseline characteristics (the first author, publication date, sample size, age range, and sex), therapeutic interventions, and results (TPOAb and TgAb titers at baseline and at endpoint). If the extracted data had any divergences, these could be assessed by a third author (Shuhang Xu). We contacted authors of included studies for missing or unclear information.

### 2.5. Data Analysis

All meta-analyses were performed using Stata statistical software (STATA version SE-10.1; Stata Corporation, College Station, TX). For each eligible study, the continuous data were presented as standardized mean difference (SMD) and 95% confidence intervals (CI). We assessed the statistical heterogeneity between trials by *I* ^2^ statistic [23]. When heterogeneity was confirmed (*P* < 0.10, *I* ^2^ > 50%), the random-effect method was used; otherwise, the fix-effect model was adopted [24]. Subgroup analyses were performed by stratifying the available data according to trial duration. A sensitivity analysis was performed to identify potential outliers. Funnel plots, Egger's test, and Begg's test were used to evaluate publication bias.

## 3. Results

### 3.1. Characteristic and Quality of Studies

**Figure 1:**
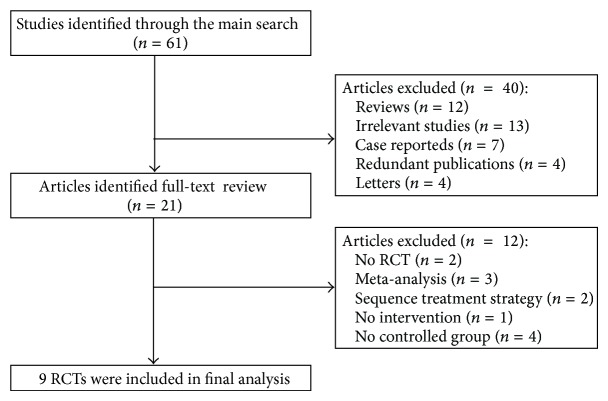
Study selection process. RCTs: randomized controlled trials.

A flow diagram of our search strategy and results is listed in Figure 1. The main search strategy identified 61 articles. By scanning titles or abstracts, 40 articles were discarded, because they were reviews, case reports, redundant publications, letters, or irrelevant studies. The full texts of the remaining 21 articles were reviewed; only 9 RCTs were eligible, and 12 articles were excluded due to two studies were non-RCT, four studies didn't have a placebo-control group or a treatment control group, three studies were meta-analyses, two studies did not state the treatment strategy, one study was not intervention study.

419 AIT patients were included in the Se supplementation group and 368 AIT patients in the placebo or no treatment group. The characteristics of the retained RCTs and the Jadad scores are shown in Table 1. The quality scores ranged from 2 to 4 points out of a theoretical maximum of 5 points. All articles adopted random assignment of patients, and 7 RCTs did not state the detailed randomized method [12, 14, 16–18, 20, 21]. The double-blinded study was performed in only 1 RCT [15]. All RCTs had defined inclusion and exclusion criteria for patients and provided clear definitions of the treatment responses.

### 3.2. The Effects of Se Supplementation on TPOAb Titers

**Figure 2:**
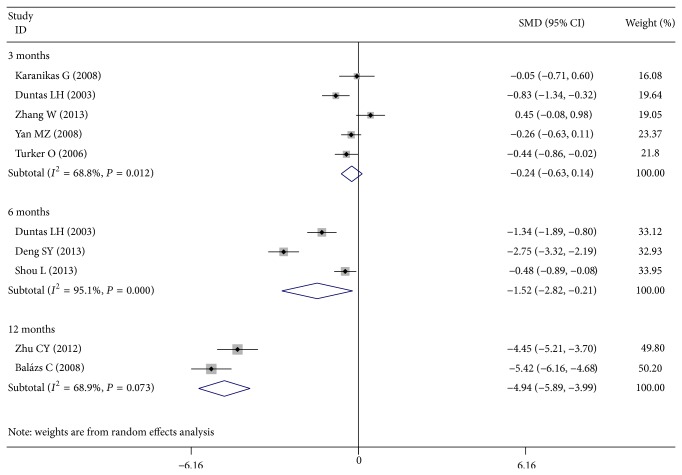
Forest plot showing the effects of Se supplementation on TPOAb titers in patients of AIT.

Six studies reported serum TPOAb titers at 3 months of treatment [12, 14, 16, 19, 20]. Patients who received Se supplementation showed no change in TPOAb titers compared with controls (SMD, −0.243; 95% CI −0.630 to 0.144; *P* = 0.218). But three studies after 6 months of treatment [16, 17, 21] and two studies after 12 months of treatment [15, 18] had different result, which showed significant lower TPOAb titers when compared with controls (6 months, SMD, −1.516; 95% CI −2.823 to −0.210; *P* = 0.023; and 12 months, SMD, −4.940; 95% CI −5.887 to −3.992; *P* < 0.001) (Figure 2).

### 3.3. The Effects of Se Supplementation on TgAb Titers

**Figure 3:**
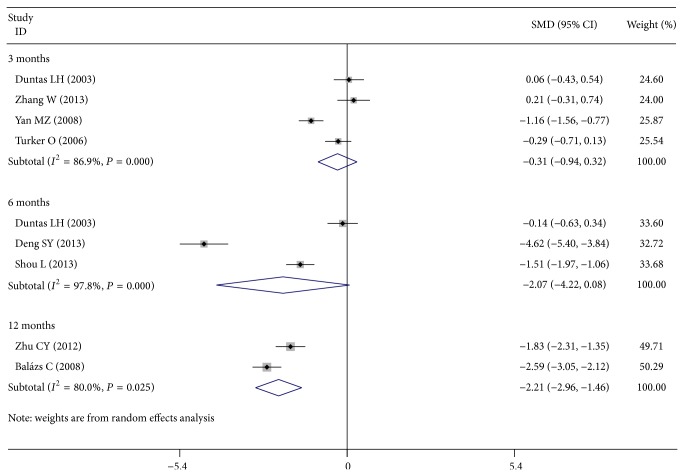
Forest plot showing the effects of Se supplementation on TgAb titers in patients of AIT.

No significant difference in TgAb titers after 3 months or 6 months of treatment was detected (four studies, 3 months, SMD, −0.310; 95% CI −0.938 to 0.319; *P* = 0.334; and three studies, 6 months, SMD, −2.068; 95% CI −4.218 to 0.081; *P* = 0.059). As compared with the control group, Se supplementation after 12 months of treatment showed significant effects on declining the TgAb titers (two studies, 12 months, SMD, −2.210; 95% CI −2.956 to −1.464; *P* < 0.001) (Figure 3).

### 3.4. The Effects of Se Supplementation on Mood

**Figure 4:**
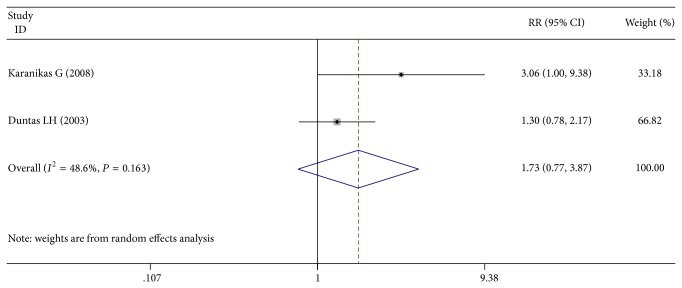
Forest plot showing the effects of Se supplementation on mood in patients of AIT.

Only two studies reported the effects of Se supplementation on mood. Patients after Se supplementation had a higher chance in improving the mood or well-being compared with controls (39/52 versus 18/49, RR = 1.61; 95% CI 1.01 to 2.57; *P* = 0.045) (Figure 4).

### 3.5. Adverse Events

Only two studies reported the side effects. One study reported that no adverse events happened [16]. Another reported that one patient suffered from gastric discomfort during Se therapy [14].

### 3.6. Publication Bias

No evidence of publication bias was found on TPOAb and TgAb titers after Se supplementation treatment, but the funnel plots for TPOAb and TgAb titers at different course of treatment were performed including a small subset of RCTs. Therefore, it is difficult to assess the results of publication bias.

### 3.7. Sensitivity Analysis

**Figure 5:**
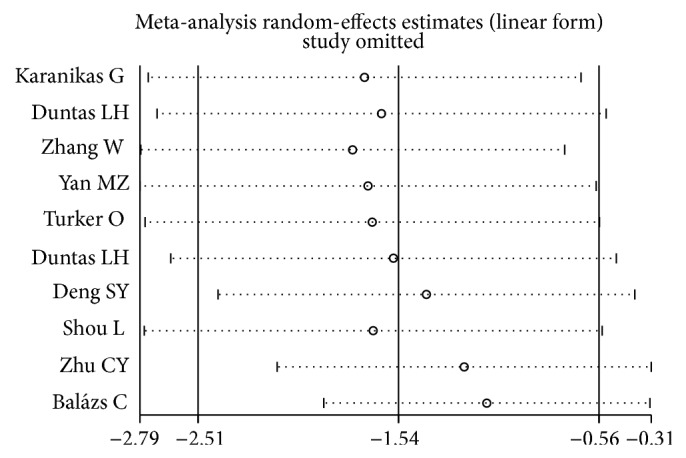
Sensitivity analysis: examining the influence of individual studies of ten studies (TPOAb titers and Se supplementation treatment). This figure shows the influence of each study on the meta-analysis, in which the meta-analysis estimates are computed by omitting one study at a time. By default, random-effects analyses are displayed.

**Figure 6:**
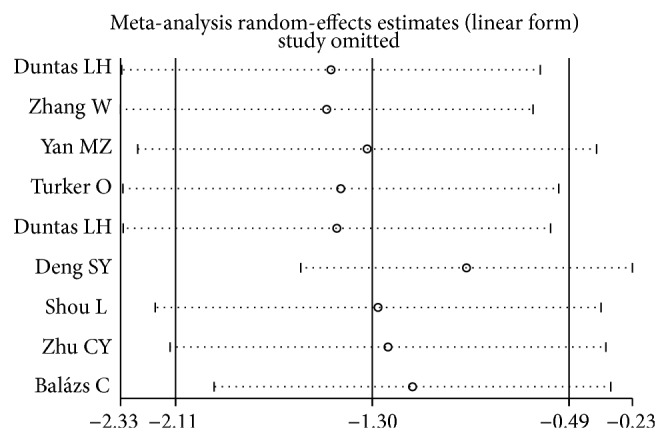
Sensitivity analysis: examining the influence of individual studies of nine studies (TgAb titers and Se supplementation treatment). This figure shows the influence of each study on the meta-analysis, in which the meta-analysis estimates are computed by omitting one study at a time. By default, random-effects analyses are displayed.

The sensitivity analysis showed that the association between the TPOAb titers and Se supplementation treatment of 10 studies (including all cases and controls) was vulnerable: when someone study was omitted at a time, the 95% CI of the model would include −1.0 (Figure 5). Further, the sensitivity analysis showed that the association between the TgAb titers and Se supplementation treatment was also vulnerable (Figure 6).

## 4. Discussion

AIT is characterized by autoimmune destruction of the thyroid [25]. Se may catalyze the extrathyroid production of T_3_ from T_4_, and Se deficiency can increase thyroid necrosis and reduce compensatory epithelial regeneration. A lot of studies have reported that Se is important for antioxidant defense and adjuvant supplementation with Se may be beneficial to AIT patients' inflammatory and immune responses [12]. Huang et al. [26] reported that Se status could affect T cell differentiation and Se deficiency is associated with Th2 cells/markers. Some articles suggested that increased Se intake may compound in AIT patients, and adequate Se status can prevent postpartum thyroiditis development [1, 27, 28]. It is well known that the pregnant women with AIT have more risk of miscarriage, preterm delivery, and development of postpartum thyroid dysfunction [29]. Some studies showed that the pregnant women taking 200 *μ*g Se during and after pregnancy were less possible of emerging some diseases when compared with untreated group [1]. However, Karanikas et al. found no immunological changes in peripheral T cells after a short period of Se supplementation [12]; this discrepancy is probably due to the immunological processes occurring in the thyroid gland in AIT.

However, the efficacy of Se supplementation for AIT patients has shown conflicting results. Our meta-analysis found that Se supplementation with duration of 6 months or 12 months significantly reduced the TPOAb titers in patients with AIT; meanwhile, Se supplementation with duration of 12 months could decrease the TgAb titers in AIT individuals. After treatment, mood improvement was found in Se supplementation group when compared with the controls. No serious adverse effects were recorded after Se supplementation, except mild gastric discomfort.

Comparing with other systematic reviews, we updated some studies about Se supplementation for AIT from 2007 to 2013 [17–21]. Our result is consistent with some meta-analysis supporting Se supplementation for AIT. Jin et al. conducted a review including 7 RCTs and had a general conclusion that Se therapy for AIT is effective and safe, though there was no change in TgAb titers [30]. However, the quantitative systematic review and meta-analysis performed by Toulis et al. reported that Se supplementation is associated with a significant decrease in TPOAb titers at 3 months (WMD: −271.09; 95% CI −421.98 to −120.19; *P* < 0.0001) [27], and this result is different from our conclusion. Gärtner et al. [31] reported that the mean TPOAb titers were significantly lower in the Se supplementation group than those in the control group after 3 months (*P* = 0.013). When the authors followed up some of the patients for 6 months, the results have no change (*P* = 0.004), but nine patients ceased this treatment and found a significant increase in their TPOAb titers [13]. The experiment carried out by Karanikas et al. in 2008 found no statistical decrease in TPOAb titers after 3 months [12]. It is possible that longer follow-up periods are needed for revealing better endpoints.

There are some limitations of our meta-analysis. Firstly, because of our strict inclusion criteria, only nine RCTs were included. These RCTs were limited by the small sample size and some studies were not double-blinded, so the results would have more bias. Secondly, we tried our best to search complete RCTs of Se supplementation for AIT, but it was affected by many uncertain factors, such as language barrier, limited retrieving resources, and publication bias. Thirdly, all studies included did not discuss the disease-process, the degree of AIT, and the forms of Se. With the aggravation of the disease, the degree of thyroid injury was getting worse steadily, and this aspect would reduce the absorption rate and the effect of Se. Meanwhile, the absorption in different forms varied. So it may lead to high heterogeneity. Last but not least, only one study has discussed the TPOAb titers. Due to their correlation with T-lymphocyte cytokine production patterns, the different TPOAb titers reflect different immunological states. It was closely associated with the immunomodulatory effects of Se on cellular immune response. So we assume that the different TPOAb titers were one of the reasons of the high heterogeneity and we will pay close attention to them. We tried to acquire data about two RCTs for all antibodies values [31, 32], but we were not able to do so. In this review, we tried to analyze the efficacy after the different follow-up time points. There were only five studies that were followed up for 3 months, three studies for 6 months, and two studies for 12 months, so it was difficult to interpret the results of publication bias due to a smaller subset of studies. Only two studies reported the adverse events after Se supplementation and two studies included the effects of Se supplementation on mood, so the definite conclusions were not possible and it should be studied in the future by higher RCTs with different follow-up time points.

In conclusion, this systematic review found the positive evidence that Se supplementation is associated with a significant decrease in TPOAb titers at 6 and 12 months; meanwhile, the TgAb titers can be dropped at 12 months. Patients after Se supplementation had a higher chance in improving the mood or well-being. Se supplementation should be one of the effective complementary treatments for AIT. More high-quality, well-designed, long term, randomized controlled, multicenter trials that are adequately powered are still needed to evaluate the real beneficial effects of the Se supplementation in AIT patients.

## Figures and Tables

**Table 1:** Characteristics of included randomized controlled trials.

Study	Sample size	Median (range) age (years)		Sex (M/F)		Intervention		Main outcome measures	Therapy period	Jadad scores
	Case/control	Case	Control	Case	Control	Case	Control			
Balázs [15]	70/62	41.4 ± 9.5	42.7 ± 8.3	2/68	1/61	L-Seleno-methionine 200 *μ*g/d	Levothyroxine	TPOAb, TgAb, TSH, FT4, FT3, urinary iodine	12 months	4

Karanikas et al. [12]	18/18	47 (19–85)		0/18	0/18	Sodium selenite 200 *μ*g/d	Placebo	TPOAb, TSH, FT4, FT3, plasma Se	3 months	2

Duntas et al. [16]	34/31	47.8 (22–61)		9/56		L-Thyroxine plus selenomethionine 200 *μ*g/d	L-Thyroxine plus placebo	TPOAb, TgAb, TSH, FT4, FT3, serum Se	3, 6 months	2

Turker et al. [14]	48/40	40.8 ± 12.5	39.2 ± 14.4	0/48	0/40	L-Thyroxine plus L-selenomethionine 200 *μ*g/d	L-Thyroxine plus placebo	TPOAb, TgAb, TSH, FT4, FT3	3 months	2

Deng et al. [17]	48/46	39 ± 12	40 ± 12	7/41	6/40	Selenious yeast tablet 200 *μ*g/d	Placebo	TPOAb, TgAb, TSH, FT4, FT3	6 months	2

Zhu et al. [18]	50/46	42.1 ± 13.6	43.4 ± 12.9	9/41	7/39	Methimazole plus selenious yeast capsule 200 *μ*g/d	Methimazole	TPOAb, TgAb, TRAb TSH, FT4, FT3	12 months	2

Zhang et al. [19]	46/20	36.3 ± 11.1	39.3 ± 13.1	4/41	1/19	L-Thyroxine plus selenious yeast tablet 200 *μ*g/d	L-Thyroxine	TPOAb, TgAb, TSH, FT4, FT3	3 months	2

Yan et al. [20]	59/55	43.8 ± 12.7	40.3 ± 11.2	8/51	6/49	L-Thyroxine plus selenomethionine 200 *μ*g/d	L-Thyroxine	TPOAb, TgAb, TSH, FT4, FT3	3 months	2

Shou et al. [21]	46/50	NM	NM	NM	NM	Sodium selenite 200 *μ*g/d	No medication	TPOAb, TgAb, TSH, FT4, FT3	6 months	2

TPOAb, thyroid peroxidase autoantibody; TgAb, thyroglobulin antibody; TSH, thyroid-stimulating hormone; FT3, free triiodothyronine; FT4, free thyroxine.
